# User-Friendly Continuous Serial Electron Diffraction Data Processing

**DOI:** 10.1063/4.0000993

**Published:** 2025-10-27

**Authors:** Paul Hager, Gerhard Hofer, Lei Wang, Laura Pacoste, Alexis Fonjallaz, Xiaodong Zou

**Affiliations:** Stockholm University, Stockholm, Stocholms län, Sweden

## Abstract

Continuous Serial Electron Diffraction (SerialED) is an emerging technique with significant potential across structural biology, chemistry, pharmaceuticals, and materials science, enabling crystal structure determination from beam-sensitive samples.[1] Yet widespread adoption remains limited, in part because data processing is perceived as complex.

We introduce a streamlined, Python–based processing suite tailored to continuous SerialED. Fast, robust preprocessing is followed by indexing and integration routines adapted from X-ray free electron laser (XFEL) data processing, and optimised to tackle the specific challenges of electron–diffraction data. The pipeline carries datasets seamlessly from raw diffraction frames to a refinement–ready HKL file, while comprehensive logging ensures full traceability and reproducibility.

A lightweight graphical interface ties the workflow together, allowing users to launch jobs, get feedback and adjust parameters without the need for scripting. By removing technical hurdles and shortening the learning curve, the package aims to lower the barrier to routine continuous SerialED across a wide range of research fields.

A beta version of the software will be provided under an MIT open-source licence.
